# PVT: An Efficient Computational Procedure to Speed up Next-generation Sequence Analysis

**DOI:** 10.1186/1471-2105-15-167

**Published:** 2014-06-04

**Authors:** Ranjan Kumar Maji, Arijita Sarkar, Sunirmal Khatua, Subhasis Dasgupta, Zhumur Ghosh

**Affiliations:** 1 Bioinformatics Centre, Bose Institute, Kolkata 700054, India; 2 Department of Computer Science and Engineering, University of Calcutta, Kolkata 700009, India; 3 Electronics and Communication Sciences Unit (ECSU), Indian Statistical Institute, Kolkata 700108, India

**Keywords:** NGS-data analysis, RNA-Seq, Cloud computing, Big data, Parallel computing, Paired end read analysis, Single end read analysis, Pipeline architecture

## Abstract

**Background:**

High-throughput Next-Generation Sequencing (NGS) techniques are advancing genomics and molecular biology research. This technology generates substantially large data which puts up a major challenge to the scientists for an efficient, cost and time effective solution to analyse such data. Further, for the different types of NGS data, there are certain common challenging steps involved in analysing those data. Spliced alignment is one such fundamental step in NGS data analysis which is extremely computational intensive as well as time consuming. There exists serious problem even with the most widely used spliced alignment tools. TopHat is one such widely used spliced alignment tools which although supports multithreading, does not efficiently utilize computational resources in terms of CPU utilization and memory. Here we have introduced PVT (Pipelined Version of TopHat) where we take up a modular approach by breaking TopHat’s serial execution into a pipeline of multiple stages, thereby increasing the degree of parallelization and computational resource utilization. Thus we address the discrepancies in TopHat so as to analyze large NGS data efficiently.

**Results:**

We analysed the SRA dataset (SRX026839 and SRX026838) consisting of single end reads and SRA data SRR1027730 consisting of paired-end reads. We used TopHat v2.0.8 to analyse these datasets and noted the CPU usage, memory footprint and execution time during spliced alignment. With this basic information, we designed PVT, a pipelined version of TopHat that removes the redundant computational steps during ‘spliced alignment’ and breaks the job into a pipeline of multiple stages (each comprising of different step(s)) to improve its resource utilization, thus reducing the execution time.

**Conclusions:**

PVT provides an improvement over TopHat for spliced alignment of NGS data analysis. PVT thus resulted in the reduction of the execution time to ~23% for the single end read dataset. Further, PVT designed for paired end reads showed an improved performance of ~41% over TopHat (for the chosen data) with respect to execution time. Moreover we propose PVT-Cloud which implements PVT pipeline in cloud computing system.

## Background

Biological systems are extremely complex and as such the information content within them is huge. Thus biological research is facing ‘big data’ problem [1]. Next-Generation Sequencing (NGS) has made this problem vastly more challenging. Today’s sequencing-based experiments provides a better understanding of complex biological systems allowing for various novel functional assays, including quantification of protein–DNA binding or histone modifications (ChIP–seq) [2], transcript levels (RNA-seq) [3], spatial interactions (using Hi-C) [4], DNA methylation modifications (MethylC-Seq) [5] and others [6]. Hence, proper interpretation of sequencing data has become particularly important. Yet such interpretation relies heavily on complex computational analysis — a new and unfamiliar domain to many of the biologists — which, unlike data generation, is not universally accessible to everyone.

NGS data analysis comprises of several steps [7]. Of these, ‘spliced alignment’ which involves alignment of the fragment (read) sequences to the reference genome, is one of the most crucial steps. During this step, the unknown reads as obtained from the sequencer are aligned with the reference genome, extended across the neighbouring aligned reads and annotated as genes, transcripts, or transcript isoform variants. This step thus reveals the identity and significance of the reads with respect to the reference genome. Moreover this is the most time-consuming and computationally resource intensive step for such analysis [8]. In this work, we have tried to put forward a solution so as to overcome the problem of longer execution time and computational resource intensiveness involved in ‘spliced alignment’ step of NGS data analysis.

Spliced alignment can be achieved using various software packages [9]. These packages can be broadly classified into two classes: Unspliced aligners like BWA [10,11], SOAP [12], Bowtie [13] and spliced aligners like GSNAP [14], QPALMA [15], AbySS [16], Trans-ABySS [17], TopHat [18]. The spliced aligners are capable of aligning reads to the reference genome as well as allows for the identification of novel splice junctions. Among the several available spliced aligners, TopHat is the most widely used alignment tool. TopHat is built on an ultrafast short read mapping program Bowtie. At first it maps the reads to the genome to obtain the potential exons. This initial mapping information provides TopHat with all the plausible splice junctions. The unmapped reads are then mapped against these plausible junctions to obtain the transcripts with annotated and novel splice junctions.

Though TopHat is one of the extensively used spliced aligners and its execution supports multithreading, it does not utilize CPU efficiently, leaves a large memory footprint during its execution and hence increases the time of execution. Moreover the alignment protocol executes certain steps repeatedly while running on individual data sets belonging to the same experiment. Such execution of redundant steps increases the execution overhead. Thus there is a scope for considerable improvement of TopHat, with regard to execution time, CPU and memory utilization without affecting the output. This can be achieved by modifying the execution workflow of TopHat. Thereby, the execution of the steps that underutilizes CPU can be run in parallel, the garbage memory can be cleared after completion of each execution step and the repetitive execution of the redundant steps can be skipped.

In this work, we have developed Pipelined Version of TopHat (PVT), wherein we take up a modular approach by breaking TopHat’s serial execution into a pipeline of multiple stages. We have implemented this modified spliced alignment execution pipeline for single end reads in a standalone system. The execution workflow for PVT utilizes CPU and memory comparatively more efficiently and reduces execution time without sacrificing the transcript annotation output of a typical TopHat run. Moreover we have customized PVT pipeline for paired end reads that ensures better performance over TopHat.

The volume of NGS dataset has reached an order of terabase and can reach upto zetabase in the near future. These massive sequencing datasets demand high-performance computational resources, rapid data transfer, large-scale data storage, and competent data analysts. This increasing volume appears to impede data mining and analysis by researchers. Hence standalone workstations will not be sufficient to handle such huge dataset. This necessitates the use of scalable, fast and computation intensive resources. A computational system known as ‘cloud’ [19], consisting of computation and data service provided via the Internet, has recently been developed. Cloud computing allows users to avail services provided by data centres without building their own infrastructure. The infrastructure of the data centre is shared by a large number of users, reducing the cost to each user. To manage the flood of NGS data, several large-scale computing framework have been implemented in cloud by Crossbow [20], Galaxy [21] and STORMSeq [22]. Implementation of TopHat in cloud cannot utilize the cloud resources efficiently due to lack of pipelined execution workflow.

Here we propose an efficient execution workflow termed as PVT-Cloud to facilitate execution of spliced alignment step in NGS analysis (both for single end and paired end reads) in a cloud computing system. This will allow low maintenance, cost effective, scalable and dynamic control of the extensive computational resource.

## Results and discussion

We designed a new execution workflow termed as PVT to expedite the ‘spliced alignment’ step in NGS data analysis. We ran PVT on the dataset containing single end reads (as given in Additional file 1: Table S1) and paired end reads (as given in Additional file 2: Table S2). We compared its performance (with respect to CPU utilization, memory utilization and execution time) with that of TopHat. Results show that PVT outperforms TopHat.

### Single end reads

#### TopHat and its pitfalls

**Figure 1:**
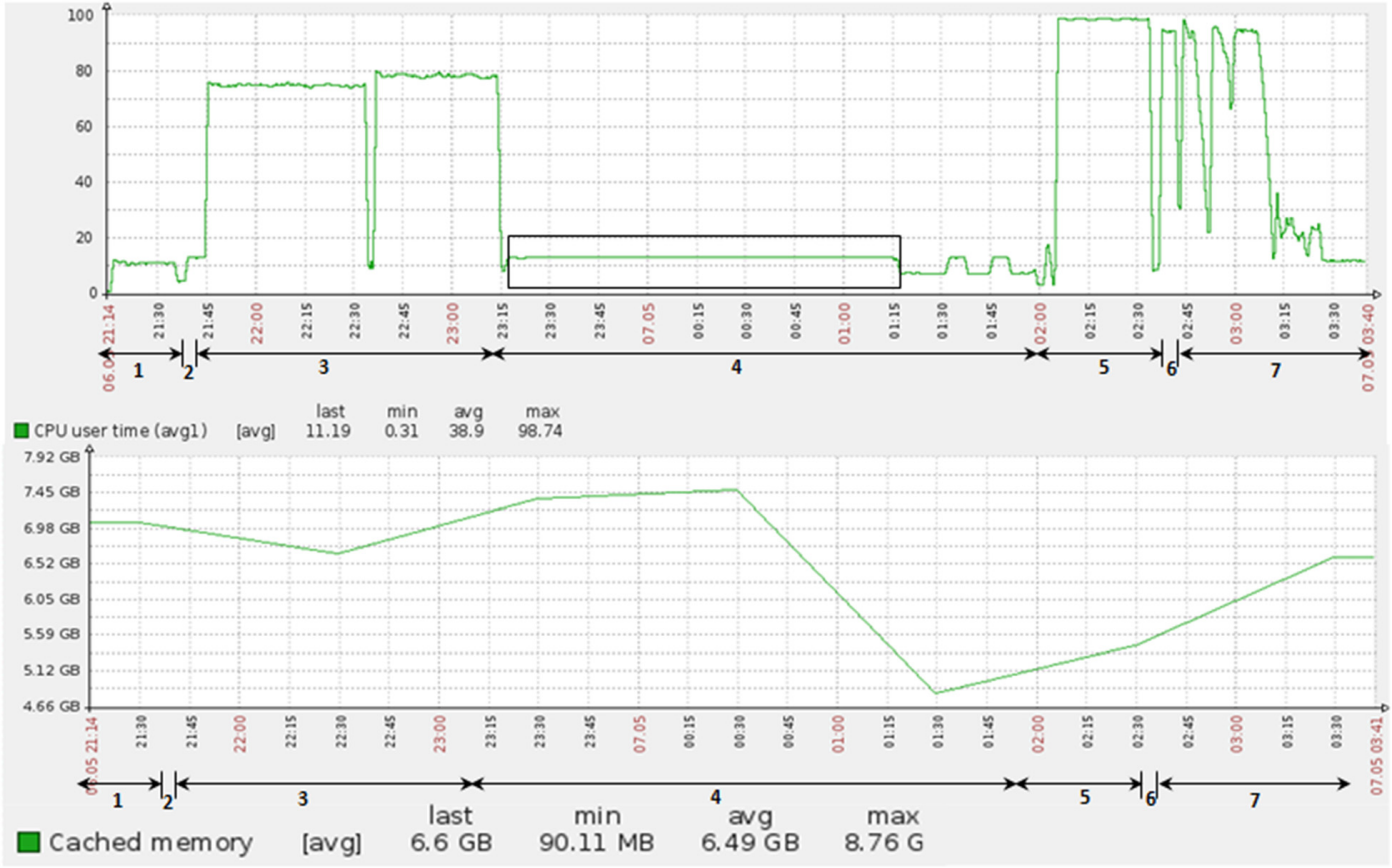
**Time (x-axis) vs performance parameters (y-axis) (CPU utilized in the top panel and cache memory in the bottom panel) during TopHat run for single end reads (SRR094775).** The standalone system has 16 GB of onboard RAM and 8 cores (3.3 GHz) CPU. Black box indicates the region where CPU is underutilized. Numbers in bold below the arrows indicate the time span of execution of different steps of TopHat: 1: *filter_reads.* 2: *gene_align.* 3: *genome_align.* 4: *find_juncs.* 5: *junc_align.* 6: *span_reads.* 7: *report.*

The different steps along with their corresponding functions involved in the ‘spliced alignment’ is discussed in details in Methods section (summarised in Additional file 3: Table S3). The order of execution of these steps during ‘spliced alignment’ using TopHat is shown in Additional file 4: Figure S1. The execution time for the single end read input dataset using TopHat is shown in Additional file 5: Figure S2. Computational resource (i.e. CPU and memory) utilization at different steps of ‘spliced alignment’ was noted for the entire single end read dataset. We have shown the result for SRR094775 data only in Figure 1, since this data consists of maximum number of filtered reads.

For the *find_juncs* step, we found that the CPU remains significantly underutilized (~12% utilization) for a considerable span of time as compared to other steps. Moreover, the average CPU utilization throughout the entire execution period for a single dataset is ~39%. Further cache memory utilized by TopHat during *find_juncs* step is also more compared to other steps These observations from the utilization graphs (Figure 1) gave us the clue that *find_juncs* is that step which can be modified to bring significant improvement in the efficiency of TopHat. Hence, parallelizing *find_juncs* would lead to an increase in CPU and memory utilization and decrease execution time. However, care should be taken towards the extent of parallelization so as to ensure no buffer overflow or CPU overutilization.

While analyzing the steps and workflow of ‘spliced alignment’, in details we found a redundant step in TopHat where the alignment tool builds the bowtie indices for aligning the reads with the genome repeatedly for processing each data file of the same experiment. This adds on to the resource consumption. These indices can be built once and used for different data files unless the reference genome is updated or a new reference organism is being worked upon. We could increase the efficiency of TopHat and reduce its execution time by building the bowtie reference indices only once for the analysis of the entire data set of an experiment.

#### PVT

**Figure 2:**
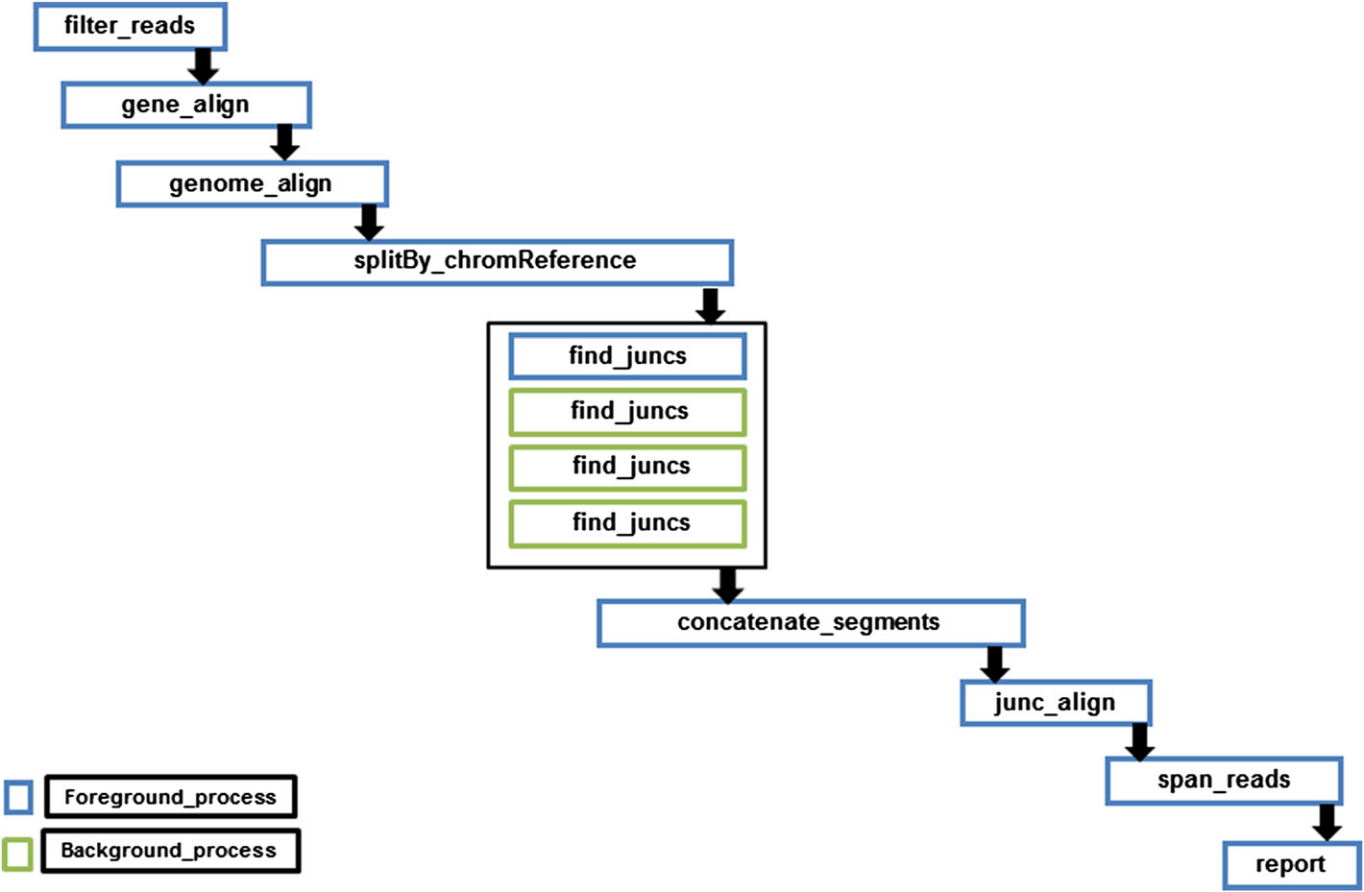
PVT pipeline and its order of execution for single end reads based on maximum CPU usage and memory utilization.

The performance analysis reports obtained on executing TopHat for single end read dataset (as shown in Figure 1) motivated us to develop PVT (Pipelined Version of TopHat) wherein we modified the execution workflow of TopHat for efficient utilization of CPU, memory and time. In PVT, the steps *filter_reads*, *gene_align* and *genome_align* are executed serially but we parallelized the *find_juncs* step so as to ensure proper utilization of computational resources, thereby reducing the execution time. Figure 2 shows the PVT alignment pipeline and its order of execution for single end reads. Rather than searching for the plausible junctions in the whole reference genome at a go, we looked for the junctions simultaneously across all reference chromosomes of the genome. Thus we were able to parallelize *find_juncs* by first splitting the reads that mapped to the genome (i.e. the output of *genome_align*), based on chromosomal reference and then concatenating the split files into comparable chromosomal reference (using **splitBy_chromReference**). The *find_juncs* is then run on separate chromosomal file references in parallel and the output of possible junctions are concatenated (using **concatenate_segments**). This is followed by serial execution of the steps- *junc_align*, *span_reads* and *report*. Parallel execution was achieved by executing the steps in both foreground and background. The blue and green bordered boxes in Figure 2 represent the processes run in the foreground and background respectively. The parallelization of this step and the execution of the subsequent steps is scheduled in such a way that no dependent step begins its execution before the completion of the previous step.

**Figure 3:**
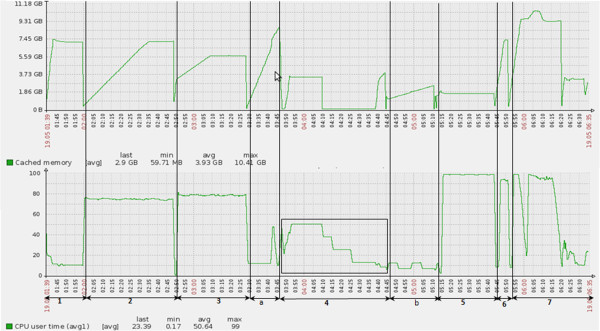
**Time(x-axis) vs performance parameters (y-axis) (cache memory in the top panel and CPU utilized in the bottom panel) during PVT run for single end read (SRR094775).** This is the result using PVT in a standalone system with 16 GB onboard RAM and 8 cores (3.3 GHz) CPU. Numbers in bold below the arrows indicate the time span of execution of different steps of PVT: 1: *filter_reads.* 2: *gene_align.* 3: *genome_align* a: *splitBy_chromReference.* 4: *find_juncs.* b: *concatenate_segments.* 5: *junc_align.* 6: *span_reads.* 7: *report.*

Prior to executing an independent step of the pipeline, clearing cache facilitates further parallelization. In PVT, the cache memory is cleared at every step to save the memory and meet the demand of other steps with high memory requirement. This helps avoid thrashing due to memory shortage and hence brings down the memory requirement for *find_juncs* (as shown in Figure 3)*.*

In PVT, we have also addressed the problem of additional resource consumption for building bowtie indices. Here, the bowtie indices were built just once for analyzing the entire dataset, thereby removing the redundancy involved in the process followed by TopHat. This indexing took ~10 minutes in our standalone configuration for the single end read analysis, which reduces 6.25% of the total execution time taken by TopHat.

*Improvement in find_juncs:* The CPU utilization during the execution of PVT pipeline for the entire single end read dataset (SRX026839 and SRX026838) was monitored. Results for SRR094775 are given in Figure 3 since this data consists of highest number of filtered reads. Results for other datasets have been provided in Additional file 6: Figure S3. The CPU utilization in the *find_juncs* increases from a meagre ~12% to approximately 50% with our modified pipeline i.e. by parallelizing the *find_juncs* (comparing Figure 1 and Figure 3). We also observed that parallelization of *find_juncs* is limited to a CPU utilization of ~50% in an 8 core CPU standalone system. Increasing the extent of parallelization to more than 4 processes with such dataset in an 8 core machine, increases the time of execution thereby putting the other processes in background to halt. However, the degree of parallelization can be extended provided *find_juncs* is executed in a standalone system having greater number of cores. Overall, the performance gain using PVT will vary depending on the volume of the input data, availability of number of CPU cores and memory of the standalone system.

#### PVT outperforms TopHat

(a) *Execution time:*

The execution time for each step involved in TopHat and PVT pipelines, run on the entire dataset was noted. As SRR094770 and SRR094775 (each chosen from the test and control data set) has larger number of filtered reads, the results for this data has been shown only (Additional file 7: Figure S4). Overall there was a significant reduction of ~23% in execution time using PVT, when compared to TopHat execution. The improvement of PVT over TopHat in *find_juncs* step for the entire single end read dataset is shown in Additional file 6: Figure S3.

**Figure 4:**
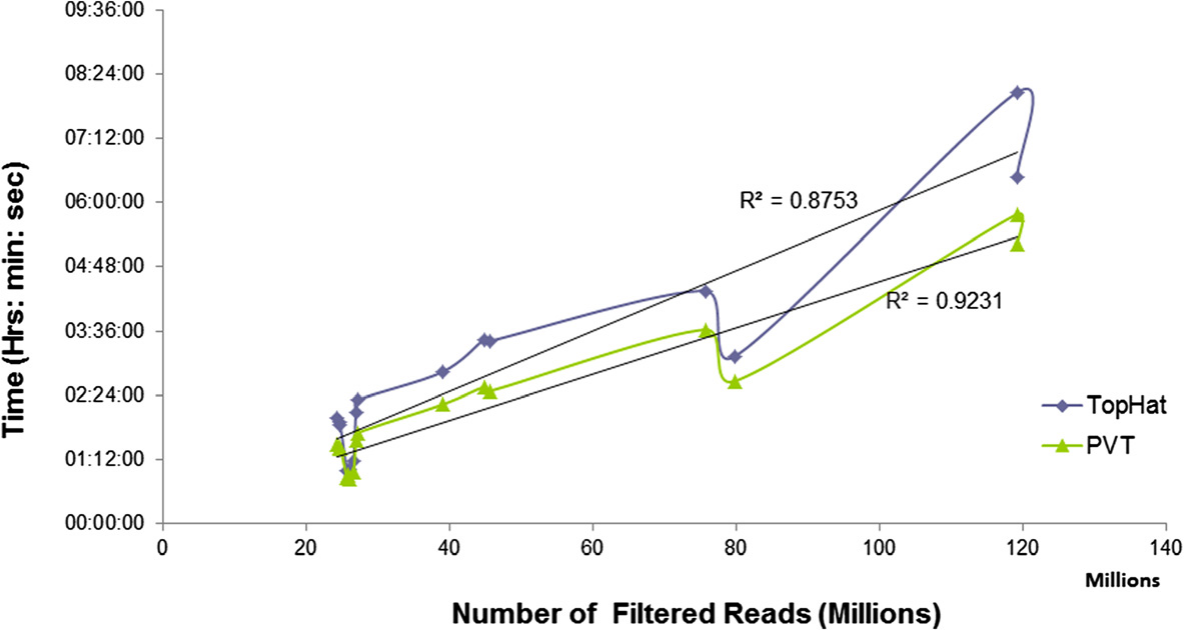
**Comparison of the trend of PVT execution time with that of TopHat for all the 16 lanes of single end reads.** Each of the curves is fitted with a linear regression line.

We compared the performance (with respect to total execution time) of PVT with that of TopHat throughout the entire ‘spliced alignment’ step (shown in Additional file 8: Figure S5) for the 16 input single end read datasets (as given in Additional file 1: Table S1). Figure 4 shows that there is a strong correlation between the number of filtered reads and the total execution time in case of both TopHat and PVT. Thus we were able to predict the execution time based on the volume of the input data (maximum prediction error was within 30% of deviation). We also assessed the prediction accuracy (prediction of execution time based on the volume of the input data) of the two pipelines by fitting a linear regression model to the curve, plotting time vs. number of filtered reads as shown in Figure 4. We obtained an R-squared value of 0.87 for TopHat whereas for PVT pipeline, R-squared value improved to 0.92. In case of TopHat there is a steep increase in the execution time when the input data size exceeds the available RAM size. This may be due to the fact that TopHat does not clear garbage memory after every step of execution thereby increasing memory thrashing. However, the steepness of the slope decreases for PVT (observed with increase in R-squared value) when we modified the execution by clearing the garbage memory at every step of the pipeline. We also observed that for smaller datasets (i.e. datasets with input size less than the available RAM size) the performance gain of PVT over TopHat was low but as the number of filtered reads increases, the performance gain increases.

(b) *CPU utilization:*

Using PVT for ‘spliced alignment’, the average CPU utilization increased from ~39% (as obtained from TopHat) to as high as ~66% for the entire single end read dataset (the average cache memory and CPU utilization for all the datasets used is given in Additional file 9: Figure S6 (A) and Additional file 9: Figure S6 (B) respectively).

### Paired end reads

#### TopHat and its pitfalls

Paired end reads (or mate pair) consist of sequence reads that occur in pairs (a constant distance of a few kb is maintained between the reads) and are obtained on sequencing both ends of the RNA simultaneously. This technique is used mainly to resolve large structural arrangements like insertions, deletions and inversions or repetitive regions of the transcriptome and is also useful for de novo transcriptome assembly. Here we modified PVT for efficient ‘spliced alignment’ of paired end reads. The dataset SRR1027730 (obtained from NCBI) for paired end reads (Additional file 2: Table S2) comprises of 45 M reads (9.5 Gbases).

**Figure 5:**
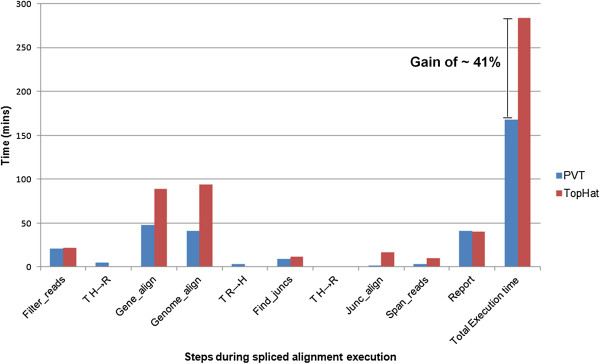
**Bar chart representing the effective time comparison between PVT and TopHat in paired end reads (SRR1027730) for different stages.** T_H → R_ indicates transferring of files from host machine to remote machine while T_R → H_ indicates the transferring of files from remote machine to host machine to be accounted for PVT execution only.

The workflow of TopHat for paired end read analysis is given in Additional file 4: Figure S1. Additional file 10: Figure S7 gives the utilization graphs (CPU utilization (%) and cache memory usage) during TopHat execution of the paired end read dataset, where the steps denoted by ‘L’ are the ones for the left kept reads and ones denoted by ‘R’ are the right kept reads. The execution time for the different steps is shown in Figure 5 for SRR1027730. The total execution time for ‘spliced alignment’ of paired end reads using TopHat takes 4 hrs 44 mins for this dataset in the specified system configurations (as mentioned in Methods Section).

On analysing the execution log file for TopHat, we observed that during the execution of the steps *gene_align*, *genome_align* , *junc_align* and *span_reads*, the left kept reads and right kept reads are processed independently of each other. These independent steps can be executed simultaneously which can reduce the entire job execution time. But TopHat performs sequential execution of the independent steps along with the dependent steps, thereby increasing the waiting time for the dependent step to begin execution. This increases the overall execution time for analysing paired end read data set using TopHat.

Moreover, TopHat breaks the read into segments. Since these segments are mapped against the reference genome (during *genome_align* execution) and against the spliced junctions (during *junc_align* execution) independently, these steps can be parallelized for both the left and right kept reads. Execution time of TopHat can be further reduced by building the bowtie reference indices only once for the analysis of the entire data set of an experiment. These motivated us to design PVT for paired-end reads where we have addressed these disadvantages.

#### PVT

In PVT for paired end reads, we scheduled the pipeline so as to run the independent jobs simultaneously on the ‘host machine’ as well as on the ‘remote machine’ so as to reduce the entire job execution time. The workflow is shown in Additional file 11: Figure S8.

The execution of the *filter_reads* step generates two output files corresponding to the left and right kept reads respectively. While running TopHat, we observed that the output containing lesser number of filtered reads (either of left kept or right kept) after executing *filter_reads* takes lesser execution time for subsequent steps as that compared to an output having higher number of reads. Again, there was a waiting time for the dependent processes to begin their execution while running TopHat. Hence, for an efficient analysis it is evident to transfer and further process the output of the step *filter_reads* containing lower number of reads to the remote machine since its transfer time (both uplink and downlink) and execution time would be less than the transfer and execution of its counterpart with higher number of reads. Thereby, the execution of the steps (*gene_align* and *genome_align; junc_align* and *span_reads*) and required transfers for the low read files, would complete simultaneously with the execution of aforementioned steps for higher read files. This will effectively reduce the waiting time for executing the dependent steps.

We denoted the output containing lower number of filtered reads by $R_{\mathrm{Low}}$ (coming from either left kept or right kept) and the other having higher number of filtered reads by $R_{\mathrm{High}}$. Hence for efficient execution of subsequent steps, $R_{\mathrm{Low}}$ is transferred to the remote machine while $R_{\mathrm{High}}$ is executed on the host machine. After *filter_reads*, the execution for $R_{\mathrm{Low}}$ and $R_{\mathrm{High}}$ can be forked into two simultaneous alignment processes executing the consecutive steps, *gene_align* and *genome_align*.

During *find_juncs*, the output of *genome_align* from both $R_{\mathrm{Low}}$ and $R_{\mathrm{High}}$ are required as input to find the spliced junctions. This requires transfer of the output generated in the remote machine (on the execution of *gene_align*, *genome_align* on $R_{\mathrm{Low}}$) to the host machine. Thus *find_juncs* is a dependent step, which waits for the two simultaneous processing of the outputs (i.e. $R_{\mathrm{Low}}$ and $R_{\mathrm{High}}$) of *genome_align* to complete. After the spliced junctions are found, the output for *R*_*Low*_ is again transferred to the remote machine for consecutive execution of the steps *junc_align* and *span_reads*. Simultaneously these steps for $R_{\mathrm{High}}$ is executed on the host machine. In the final step *report*, the aligned output obtained from $R_{\mathrm{Low}}$ and $R_{\mathrm{High}}$ after execution of the previous steps (as mentioned above) can be concatenated, thereby generating the final alignment output and possible spliced junctions.

Theoretically the total time required for the execution of PVT and TopHat (equation (1a) and (1b)) for paired end reads can be calculated as follows:

(1a) $$T_{i\_\mathrm{PVT}}=\max\left[\left(t_{T\to R_{\mathrm{Low}}}+t_{ER_{\mathrm{Low}}}+t_{T\leftarrow R_{\mathrm{Low}}}\right),t_{ER_{\mathrm{High}}}\right]$$

(1b) $$T_{i\_\mathrm{TopHat}}=t_{ER_{\mathrm{Low}}}+t_{ER_{\mathrm{High}}}$$

Here *i* denotes the sets of independent steps executed simultaneously in the host machine and remote machine. For *i=1*, the set of independent consecutive steps to be executed are *gene_align* and *genome_align* and for *i=2*, the set of independent consecutive steps to be executed are *junc_align* and *span_reads*.

$$\begin{array}{ll} T_{i\_\mathrm{PVT}} & =\mathrm{the}\;\mathrm{effective}\;\mathrm{time}\;\mathrm{required}\;\mathrm{for}\;\mathrm{completion}\\ & \;\mathrm{of}\;\mathrm{each}\;\mathrm{independent}\;\mathrm{steps}\;\mathrm{of}\;\mathrm{PVT} \end{array}$$

$$\begin{array}{ll} T_{i\_\mathrm{TopHat}} & =\mathrm{the}\;\mathrm{effective}\;\mathrm{time}\;\mathrm{required}\;\mathrm{for}\;\mathrm{completion}\\ & \;\mathrm{of}\;\mathrm{each}\;\mathrm{independent}\;\mathrm{steps}\;\mathrm{of}\;\mathrm{TopHa}t \end{array}$$

$$\begin{array}{ll} t_{T\to R_{\mathrm{Low}}} & =\mathrm{transfer}\;\mathrm{time}\;\mathrm{of}\;R_{\mathrm{Low}}\;\mathrm{to}\;\mathrm{the}\;\mathrm{remote}\;\mathrm{machine}\\ & \;\mathrm{for}\;\mathrm{executing}\;\mathrm{each}\;\mathrm{set}\;\mathrm{of}\;\mathrm{independent}\;\mathrm{steps} \end{array}$$

$$\begin{array}{ll} t_{ER_{\mathrm{Low}}} & =\mathrm{execution}\;\mathrm{time}\;R_{\mathrm{Low}}\;\mathrm{in}\;\mathrm{the}\;\mathrm{remote}\;\mathrm{machine}\\ & \;\mathrm{for}\;\mathrm{each}\;\mathrm{set}\;\mathrm{of}\;\mathrm{independent}\;\mathrm{steps} \end{array}$$

$$\begin{array}{ll} t_{T\leftarrow R_{\mathrm{Low}}} & =\mathrm{transfer}\;\mathrm{time}\;\mathrm{of}\;R_{\mathrm{Low}}\;\mathrm{from}\;\mathrm{remote}\;\mathrm{machine}\\ & \;\mathrm{after}\;\mathrm{execution}\;\mathrm{of}\;\mathrm{each}\;\mathrm{set}\;\mathrm{of}\;\mathrm{independent}\;\mathrm{steps} \end{array}$$

$$\begin{array}{ll} t_{ER_{\mathrm{High}}} & =\mathrm{execution}\;\mathrm{time}\;R_{\mathrm{High}}\;\mathrm{in}\;\mathrm{the}\;\mathrm{home}\;\mathrm{machine}\\ & \;\mathrm{for}\;\mathrm{each}\;\mathrm{set}\;\mathrm{of}\;\mathrm{independent}\;\mathrm{steps} \end{array}$$

Thereby, the total execution time $T_{\mathrm{Total}\_\mathrm{PVT}}$ (for PVT) and $T_{\mathrm{Total}\_\mathrm{TopHat}}$ (for TopHat) is given as follows:

(2a) $$T_{\mathrm{Total}\_\mathrm{PVT}}=T_{\mathrm{FR}}+\sum_{i=1}^{2}T_{i\_\mathrm{PVT}}+T_{\mathrm{FJ}}+T_{R}$$

(2b) $$T_{\mathrm{Total}\_\mathrm{TopHat}}=T_{\mathrm{FR}}+\sum_{i=1}^{2}T_{i\_\mathrm{TopHat}}+T_{\mathrm{FJ}}+T_{R}$$

Here

$$T_{\mathrm{FR}}=\mathrm{time}\;\mathrm{contribution}\;\mathrm{from}\;\mathrm{the}\;\mathrm{step}\;\mathrm{filter}\_\mathrm{reads}$$

$$\sum_{i=1}^{2}T_{i\_\mathrm{PVT}}=\;\mathrm{time}\;\mathrm{contribution}\;\mathrm{from}\;\mathrm{the}\;\mathrm{two}\;\mathrm{sets}\;\mathrm{of}\;\mathrm{independent}\;\mathrm{steps}\;\mathrm{defined}\;\mathrm{above}\;\mathrm{for}\;\mathrm{PVT}.$$

$$\begin{array}{ll} \sum_{i=1}^{2}T_{i\_\mathrm{TopHat}} & =\mathrm{time}\;\mathrm{contribution}\;\mathrm{from}\;\mathrm{the}\;\mathrm{two}\;\mathrm{sets}\;\mathrm{of}\\ & \;\mathrm{independent}\;\mathrm{steps}\;\mathrm{defined}\;\mathrm{above}\;\mathrm{for}\;\mathrm{TopHat}. \end{array}$$

$$T_{\mathrm{FJ}}=\mathrm{time}\;\mathrm{contribution}\;\mathrm{from}\;\mathrm{the}\;\mathrm{step}\;\mathrm{find}\_\mathrm{juncs}$$

$$T_{R}=\mathrm{time}\;\mathrm{contribution}\;\mathrm{from}\;\mathrm{the}\;\mathrm{step}\;\mathrm{report}$$

The improvement (in percentage) in the total execution time of PVT over that of TopHat can be given by

(3) $$\frac{T_{\mathrm{Total}\_\mathrm{TopHat}}-T_{\mathrm{Total}\_\mathrm{PVT}}}{T_{\mathrm{Total}\_\mathrm{TopHat}}}\times 100$$

Using the above equations (1) and (2), we theoretically estimated the total PVT execution time for SRR1027730 based on TopHat execution time needed for each step. We obtained all the execution times $\left(t_{ER_{\mathrm{Low}}},t_{ER_{\mathrm{High}}},T_{\mathrm{FR}},T_{\mathrm{FJ}}\;\mathrm{and}\;T_{R}\right)$ from TopHat execution times noted for paired end read analysis. The transfer times in PVT $\left(t_{T\to R_{\mathrm{Low}}}\;\mathrm{and}\;t_{T\leftarrow R_{\mathrm{Low}}}\right)$ for the required inputs and outputs (for the different sets of independent steps) using *scp* were noted for the corresponding inputs and outputs in TopHat.

Substituting the execution time required for each step using TopHat and the transfer times for the independent steps (given in Table 1) in equations (1), (2) and (3),we obtained a significant reduction of ~34% in execution time using PVT for SRR1027730, when compared to that using TopHat.

**Table 1:** Comparison of the time of execution for the paired end reads (SRR1027730) using TopHat and PVT pipeline in the host and remote machines respectively

**Steps**	**TopHat**	**PVT**		
	**Execution Time (mins)**	**Execution time (mins) in Host machine**	**Execution time (mins) in Remote machine**	**Time (mins) considered for PVT of paired end reads**
*Filter_reads*	22	21	-	21
*Transfer to remote*	Not applicable	5		92*
*Building Bowtie indices*	6	6		
*Gene_align*	83	42(L)	37(R)	
*Genome_align*	94	39(L)	41(R)	
*Transfer from remote*	Not applicable	3		
*Find_juncs*	12	9	-	9
*Transfer to remote*	Not applicable	Negligible (~22 s)		5*
*Junc_align*	17	2(L)	2(R)	
*Span_reads*	10	3(L)	3(R)	
*Transfer from remote*	Not applicable	~24 s		
*Report*	40	41(L + R)	-	41
*Net execution time*	*284*	168		

(L) and (R) denotes the execution time for left and right kept reads respectively.

*Time calculated using equation (1).

In the experiment, we implemented the PVT pipeline for SRR1027730 in two similar standalone configurations and obtained the time duration for each of the steps as given in Table 1. The improvement of PVT over TopHat as observed for the same data (shown in Additional file 12: Figure S9) experimentally was ~41% which is more than that as obtained by theoretical calculation i.e. ~34%. Such added improvement might be due to additional modification of PVT over TopHat which has been done by parallelizing sub-steps of *genome_align* and *junc_align*. This time reduction couldn’t be taken into account while calculating performance improvement of PVT over TopHat theoretically.

### PVT- Pipeline setup for processing multiple data files

Processing of multiple data files (with SRR IDs) in an experiment consists of repeated computation for spliced alignment. Hence, pipelined execution of spliced alignment increases the *speedup*. PVT enables implementation of the execution workflow as a pipeline, consisting of multiple stages [23]. Each stage can work on different execution steps at the same time thus requiring the pipeline to be run on separate instance(s)/standalone configurations.

In PVT we have defined five *stages* (given in Additional file 13: Table S4) based on the steps executed for spliced alignment (Figure 2 for single end reads and Additional file 11: Figure S8 for paired end reads). We have selected the steps in each stage so that there is balanced length of pipeline stages which will increase the *speedup*. Based on our analysis of execution time for both single end read and paired end reads (Figure 3 and Additional file 12: Figure S9), we merged the *steps* to build each *stage* of the pipeline as shown in Additional file 11: Figure S8.

As Stage II, (consisting of *genome_align*) is most time consuming, we can choose a larger instance/higher machine configuration or a cluster of instances to bring down the execution time of this stage to a comparable length as that of the other stages in the pipeline. Thus, we are able to overlap the execution of multiple data files i.e. when a job is executed in a particular instance(s)/standalone configuration(s) (executing a specific stage of the pipeline), the next submitted job is executed in another instance(s)/standalone configuration(s) (executing the previous stage of the pipeline).

**Figure 6:**
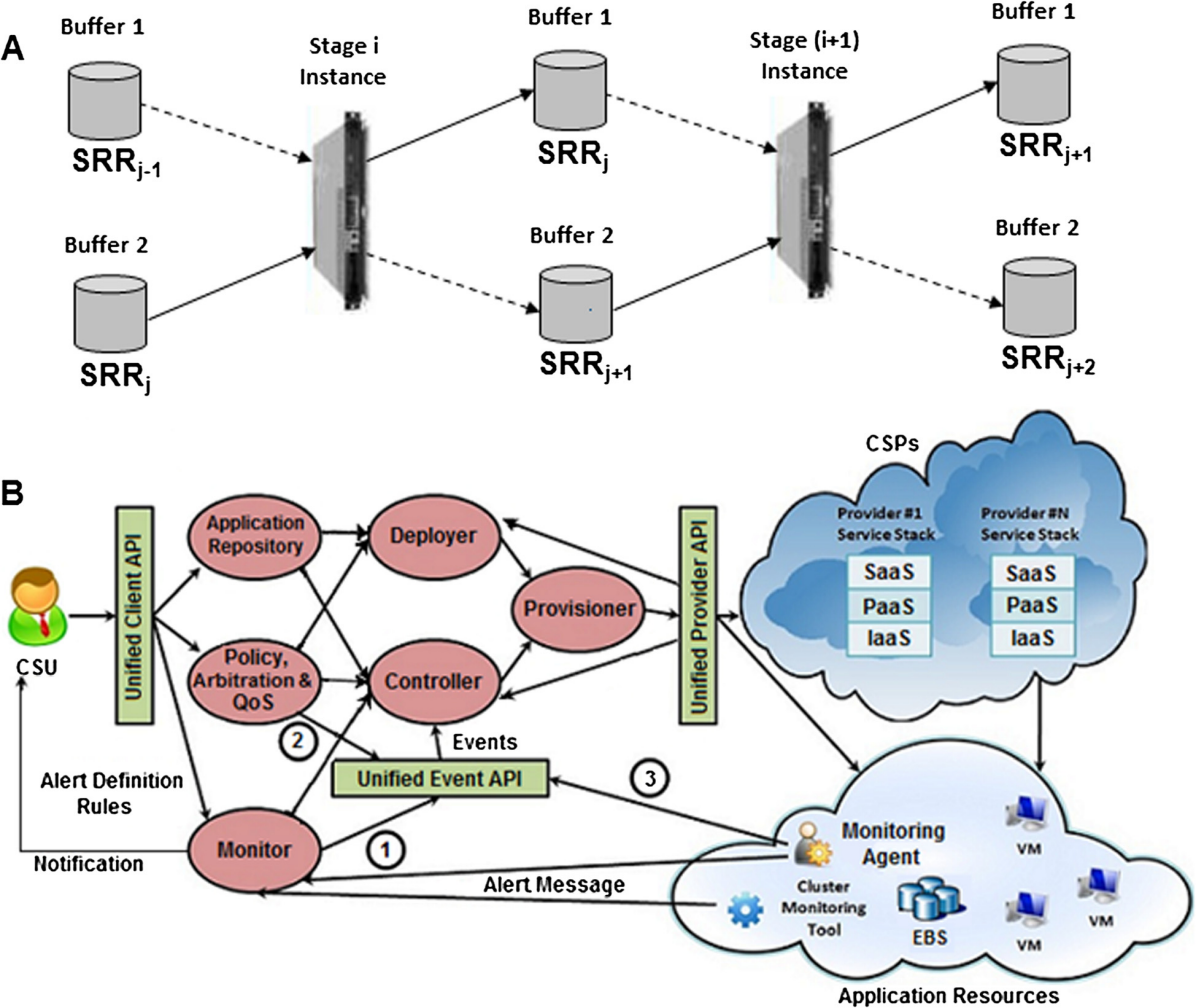
**(A): Pipeline buffer management for two consecutive stages of PVT (** ⇢ **denotes the buffer mounted in the even time slots,** ⇢ **denotes the buffer mounted in the odd time slots). (B): Middleware based cloud architecture which is based on MOVR proposed by Khatua et al. [**[24]**].**

The stages of the PVT pipeline have different delays. Hence we need to put appropriate buffers (storage) in between the stages to synchronize their executions. Since a buffer can be mounted to a single instance at a time, it will create problem for parallel execution of the consecutive stages in the PVT pipeline. To solve this, we can use two buffers and mount them to the consecutive stages in an alternate fashion as shown in Figure 6(A). Considering a particular instance executing stage i of the pipeline, instance (i + 1) executes its next stage. While executing multiple SRR files submitted in the job queue in descending order, the head of the queue has the higher job number, Thus the job with higher number i.e. SRR_j+1_ will be called in for execution before the job having lower job number SRR_j-1_. The buffers are mounted to the instances as shown by the solid lines at every even time slots and by the dotted lines at every odd time slots of the pipeline.

The PVT pipeline, described above, does not suffer from any kind of pipeline hazards [23] since PVT stages do not have any dependency on each other. Overall, there will be a significant reduction in execution time in PVT as compared to that in TopHat.

The dynamic reservation of the instances and set up of the pipeline buffers as discussed above, can be faithfully implemented in the cloud computing system which provides an efficient and manageable architecture for PVT.

### PVT-Cloud: pragmatic cloud architecture

The presence of huge sequence data for alignment analysis, requires an extensive computational resource. Although TopHat in multithreaded mode can be implemented in cloud, it would reserve the instances for a longer duration of time, increasing the reservation cost and hence is incapable of taking the advantage of such an extensive computational resource to process extensive dataset. Direct implementation of TopHat in cloud fails to utilize the elastic feature of cloud resources. As PVT is able to overlap the execution of multiple data files and each pipeline stage (as given in Additional file 13: Table S4) works on a different execution step at the same time, it brings upon a huge improvement compared to TopHat. Here we propose an architecture termed as PVT-Cloud which can be implemented in a middleware based cloud architecture (in Figure 6(B)) based on that as proposed by Khatua et al. [24].

We have defined an application as a set of NGS data, each of which is specified by an URL to corresponding databases. The end user submits such an application to application repository for analysing each NGS data using PVT. The end user may set their policy, QoS (Quality of Service) etc. to control analysis of their NGS data, if required. The *deployer* module sets up the pipeline for PVT and finds the initial optimal resources for the PVT and the QoS provided. Once the PVT is setup, either in a standalone or cluster system, the required monitoring agents are automatically installed within the deployed resources. The monitoring agents send the status of the resources as well as PVT execution dynamically to the *monitor* module. The *monitor* module correlates the information sent by the agents to generate *events* for the *controller* module. Each event designates a significant change (e.g. CPU over provisioned, CPU under used, memory over provisioned, memory under used, completion of a PVT stage etc.) required in the PVT execution. Once an event is received, the *controller* module takes the necessary action to optimize the current stage of PVT execution. For example, at the completion of a PVT stage, the *controller* module will schedule the current sequence data to the next stage while allocating the current stage to the next submitted sequence data. The *monitor* module sends notification to the user on successful completion of analysis of the submitted dataset. In this way, analysis of multiple NGS data will be carried out concurrently within PVT using a limited amount of resources.

Presently, due to lack of resources, we are unable to show the performance of pipelined execution of PVT-Cloud.

## Conclusion

NGS data helps to understand the biomolecular interactions in depth. In order to analyze such large volume of data with high degree of accuracy, an efficient protocol is necessary that improves computational resource utilization. These days biologists are facing problems to manage ‘big data’. This demands better and enhanced insight into various NGS data. In any kind of sequence data analysis, alignment to the reference genome is the most important step to annotate and extract the significance of the read. TopHat is the most widely used spliced alignment tool that determines transcript variants for both novel and annotated ones based on the alignment of the read sequences with the reference genome.

In this work we modified the TopHat workflow for both single end and paired end reads in order to increase its efficiency with respect to its computation time and computational resource utilization (in terms of CPU and memory utilization). In PVT for single end reads, we parallelized the steps where the computational resource is underutilized and removed the redundant steps during the execution of each dataset which improved its efficiency and enforced utilization of computational resource along with the reduction of the execution time. For paired end reads we rescheduled the execution of each steps and distributed the job in separate machines, in addition to removing the redundant steps during the execution of each dataset. For single end read analysis, PVT resulted in reduction of the execution time to ~23% as compared to TopHat, whereas for paired end read analysis the execution time reduced to ~41%. Further, we proposed a cloud architecture PVT-Cloud for running single end and paired end reads in cloud for a time effective method of processing NGS data. Our modified protocol thus increases the degree of parallelization, computational resource utilization and thereby reduces the execution time in both standalone and distributed system architecture.

Overall, our approach suggests betterment towards executing the spliced alignment step efficiently with a significant reduction in execution time and proper utilization of computational resources. PVT in cloud system ensures better performance than that in a standalone system. Implementing PVT will speed up the execution process and will provide a cost-effective solution for NGS data analysis.

## Methods

### Input data set

We downloaded the sequence data corresponding to single end and paired end reads from NCBI (http://www.ncbi.nlm.nih.gov/) for single end and paired end reads (Additional file 1: Table S1 and Additional file 2: Table S2) which are as follows:

*For single end reads*: The data for single end reads was downloaded from NCBI (SRA database: Accession Number: SRX026839 and SRX026838). The dataset corresponds to mRNA sequence reads (transcripts) of adipose derived induced pluripotent stem cells (ADS_iPSC) test sample (SRX026838-test sample) and human embryonic stem cell (hESC) control sample (SRX026839). Each test and control sample has 8 runs with a total of 308.9 M reads (13.2 Gbases) and 446.9 M reads (19.2 Gbases) respectively. Corresponding reference annotations for the human genome were downloaded from NCBI (build 37/hg19) [*http://www.ncbi.nlm.nih.gov*].

*For paired end reads*: The SRR1027730 was downloaded from NCBI which comprises of 45 M reads (9.5 Gbases). Corresponding reference annotations are the same as that used for single end reads. The dataset corresponds to mRNA sequence reads of pancreatic islets.

### Standalone configuration

*For single end reads:* The analysis for single-end reads was carried out in a standalone system with an Intel Xeon E3-1245 8 core processor, 16GB RAM 64 bit and a processor speed of 3.3 GHz with Ubuntu. We ensured no other processes were running in the background (450 MB memory consumption for init processes) except ZABBIX-client (http://www.zabbix.com/) to monitor resource utilization. The ZABBIX server was configured on another system which collected the performance report for TopHat and PVT.

*For paired end reads:* The analysis for paired-end reads was carried out in two standalone systems:

(a) *Host machine*: Intel Xeon E3-1245 8 core processor, 16GB RAM 64 bit and a processor speed of 3.3 GHz with Ubuntu.

(b) *Remote machine:* Intel Xeon E3-1245 8 core processor, 16GB RAM 64 bit and a processor speed of 3.4 GHz with Ubuntu.

### Processing the reads

The archived reads were decompressed using SRA-Toolkit 2.1.10 (http://eutils.ncbi.nih.gov/Traces/sra/sra.cgi?view=software) and spliced alignment was carried out with TopHat v 2.0.8. *Bowtie* 2.1.0 was used for short read alignment and *Samtools* 0.1.18 [25] was used for conversion between various alignment formats and processing of the alignment outputs.

We ran the spliced alignment tool using TopHat v 2.0.8 on the entire data set for both single and paired end reads in the above mentioned standalone configuration. The time of execution for each run was noted. The CPU and memory performance were constantly monitored during the execution period using ZABBIX.

### TopHat

A typical TopHat execution comprises of steps as given in Additional file 3: Table S3 (along with its abbreviations and functions of each step). The dataflow for the execution is shown in Additional file 4: Figure S1. In the first step (*filter_reads*), TopHat prepares the sequence reads for alignment by filtering out the low quality score reads (in *fastq* format). It then aligns the filtered reads to the set of reference genes (exonic sequences) using the short read aligner *bowtie* in the *gene_align* step*.* The unmapped reads from the *gene_align* step are aligned with the reference genome in *genome_align* step which returns the set of mapped reads and the initial set of unmapped reads. The initial mapping information is used to build a database of possible splice junctions in the *find_juncs* step. The segments which did not align with the reference genome (i.e. in *genome_align* step) are then mapped against these possible spliced junctions (*junc_align*). During *span_reads* the aligned segments are spanned across the neighbouring exon alignments to form the novel and annotated transcripts according to the splice junctions detected. In the final step *report,* all the unaligned and the aligned reads are finally reported along with the potent junctions. For paired end reads, the TopHat execution step is the same; just that the left and right kept reads are processed separately.

### PVT

*For single end reads*: The PVT pipeline for single end reads consists of the following steps (flow diagram shown in Figure 2). Input read sequences are filtered based on quality scores (*filter_reads*). The filtered reads are then aligned with the gene annotations downloaded from NCBI using Bowtie (*gene_align*). The unmapped reads are further aligned with the genome annotations downloaded from NCBI (*genome_align*). The reads that mapped with the genome were split based on chromosomal references (using **splitBy_chromReference**) to enable forking of the *find_juncs* step. This enables us to parallelize the step and increase the efficiency of computational resource usage. This step is followed by concatenation of segments (using **concatenate_segments**) where the sequences of the possible junctions are concatenated The junctions are then aligned with the reads that mapped with the genome (*junc_align*), the output being the possible spliced junctions. The *span_reads* step then extends the neighbouring reads that mapped to the genome to obtain the spliced reads. Finally the *report* step provides the output containing the accepted alignments that are possible with the read sequences, along with the unaligned reads and the potent junctions. A pseudo-code for the execution of the PVT pipeline for single end reads is given in Additional file 14: Table S5. Overall, PVT increases the degree of parallelization for the less CPU utilized step to achieve efficient resource utilization. Moreover to reduce the total execution time, we removed the redundant steps that are executed repetitively, thereby reducing the execution overhead.

*For paired end reads*: A flow diagram of PVT pipeline for the paired end reads is shown in Additional file 11: Figure S8. Here, we schedule the execution of the independent steps comprising the entire execution pipeline in such a manner, so that the job can be distributed into multiple systems, thereby reducing the execution time significantly. All the steps here are similar as those described in for single end reads, except that the steps *gene_align*, *genome_align*, *junc_align* and *span_reads* are executed independently for the left and right kept reads in host and remote machines. Moreover, mapping of the segments against the reference genome (during *genome_align* execution) and against the spliced junctions (during *junc_align* execution) are parallelized for both the left and right kept reads. A pseudo-code for the execution of the PVT pipeline for paired end reads is given in Additional file 15: Table S6.

## Competing interests

The authors declare that they have no competing interests.

## Authors’ contributions

RM and AS: conception and design, collection and/or assembly of data, data analysis and interpretation, manuscript writing, SK and SD: design and assembly of data, ZG: conception and design, data analysis and interpretation, drafting the manuscript and revising it critically for important intellectual content. All authors read and approved the final manuscript.

## Supplementary Material

Additional file 1: Table S1 Description of the input files downloaded from NCBI (SRA database: Accession Number: SRX026839 and SRX026838) for single end read analysis (A) corresponds to mRNA sequence reads of human embryonic stem cell (hESC)-control sample, (SRX026839) (B) corresponds to mRNA sequence reads of adipose derived induced pluripotent stem cells (ADS_iPSC)-test sample. Click here for file

Additional file 2: Table S2 Description of the input file for paired end reads (SRR1027730) downloaded from NCBI. The data corresponds to mRNA sequence reads of pancreatic islets. Click here for file

Additional file 3: Table S3 Sequential steps for ‘spliced alignment’ in NGS data analysis. Click here for file

Additional file 4: Figure S1 TopHat pipeline and its order of execution both for single end and paired end reads ( ⇢ indicates unmapped outputs, → indicates mapped outputs). Click here for file

Additional file 5: Figure S2 TopHat Execution time corresponding to the entire single end read dataset (SRX026839 and SRX026838). Click here for file

Additional file 6: Figure S3 Improvement of PVT over TopHat for the entire single-end read dataset in the *find_juncs* step. Click here for file

Additional file 7: Figure S4 Time comparison between TopHat and PVT for all the sub-steps for (A) SRR094770 (B) SRR094775. Click here for file

Additional file 8: Figure S5 Bar Graph representing the comparison of PVT execution time with that of TopHat for the entire single end read dataset. Click here for file

Additional file 9: Figure S6 Comparison of (A) Cache memory (in GB) utilization (B) average CPU utilization (in %) for the entire single end read dataset. Click here for file

Additional file 10: Figure S7 Time(x-axis) vrs (A) CPU utilized (B) cache memory utilized in a standalone system with 16 GB of onboard RAM and 8 cores (3.3 GHz) CPU during the run for paired end read (SRR1027730) using TopHat. Abbreviations indicated in bold below arrow denotes the different steps of execution: L- left kept reads, R- right kept reads, numbers- denotes the step number 1: *filter_reads.* 2: *gene_align.* 3: *genome_align.* 4: *find_juncs.* 5: *junc_align.* 6: *span_reads.* 7: *report.* Click here for file

Additional file 11: Figure S8 PVT pipeline showing the order of execution and different stages for single end and paired end reads. The step(s) comprising each stage is based on balanced length of pipeline stage. Single end read analysis pipeline is presented within black bordered box. Click here for file

Additional file 12: Figure S9 Time (x-axis) vs (A) CPU utilized in the host machine (B) CPU utilized in the remote machine (C) cache memory utilized in the host machine and (D) cache memory utilized in the remote machine during the run for paired end reads (SRR1027730) using PVT. Abbreviations indicated in bold below arrow denotes the different steps of execution: L- left kept reads, R- right kept reads, numbers- denotes the step number 1: *filter_reads.* 2: *gene_align.* 3: *genome_align.* 4: *find_juncs.* 5: *junc_align.* 6: *span_reads.* 7: *report.* Click here for file

Additional file 13: Table S4 Spliced alignment steps corresponding to each pipeline stage. Click here for file

Additional file 14: Table S5 Pseudo-code of PVT for single end read analysis. Click here for file

Additional file 15: Table S6 Pseudo-code of PVT for paired end read analysis. Click here for file
