# Supplementary material for: PVT: An Efficient Computational Procedure to Speed up Next-generation Sequence Analysis
Source: BMC Bioinformatics. 2014 Jun 4;15:167. doi: 10.1186/1471-2105-15-167 (PMC4063226; doi:10.1186/1471-2105-15-167)
Supplement: Additional file 1: Table S1 — Description of the input files downloaded from NCBI (SRA database: Accession Number: SRX026839 and SRX026838) for single end read analysis (A) corresponds to mRNA sequence reads of human embryonic stem cell (hESC)-control sample, (SRX026839) (B) corresponds to mRNA sequence reads of adipose derived induced pluripotent stem cells (ADS_iPSC)-test sample. [file 1471-2105-15-167-S1.doc]

Supplementary Table 1:

| **A**  **Accession: SRX026839 CONTROL: hESC** | | |
| --- | --- | --- |
| **Run** | **# of Reads** | **# of Bases** |
| SRR094768 | 44,845,518 | 1.9G |
| SRR094769 | 27,239,445 | 1.1G |
| SRR094770 | 119,155,920 | 5G |
| SRR094771 | 45,701,495 | 1.9G |
| SRR094772 | 39,042,945 | 1.6G |
| SRR094773 | 24,818,985 | 1G |
| SRR094774 | 26,948,827 | 1.1G |
| SRR094775 | 119,167,795 | 5G |
