# Supplementary material for: PVT: An Efficient Computational Procedure to Speed up Next-generation Sequence Analysis
Source: BMC Bioinformatics. 2014 Jun 4;15:167. doi: 10.1186/1471-2105-15-167 (PMC4063226; doi:10.1186/1471-2105-15-167)
Supplement: Additional file 2: Table S2 — Description of the input file for paired end reads (SRR1027730) downloaded from NCBI. The data corresponds to mRNA sequence reads of pancreatic islets. [file 1471-2105-15-167-S2.doc]

**Supplementary Table2:**

| **Accession: SRX375277** | | |
| --- | --- | --- |
| **Run** | **# of Reads** | **# of Bases** |
| SRR1027730 | 45,748,159 | 9.2G |
