# Supplementary material for: PVT: An Efficient Computational Procedure to Speed up Next-generation Sequence Analysis
Source: BMC Bioinformatics. 2014 Jun 4;15:167. doi: 10.1186/1471-2105-15-167 (PMC4063226; doi:10.1186/1471-2105-15-167)
Supplement: Additional file 3: Table S3 — Sequential steps for ‘spliced alignment’ in NGS data analysis. [file 1471-2105-15-167-S3.doc]

Supplementary Table 3:

| **Sl. No.** | **Steps** | **Abbreviated notations** | **Functions** |
| --- | --- | --- | --- |
| 1 | Filtering reads | *filter_reads* | prepares the sequence reads for alignment by filtering out the low quality score reads |
| 2 | Gene alignment | *gene_align* | aligns the filtered reads to the set of reference genes using the short read aligner *bowtie* |
| 3 | Genome alignment | *genome_align* | unaligned reads are mapped with the reference genome |
| 4 | Finding_junctions | *find_juncs* | builds a database of possible splice junctions using the initially unmapped reads |
| 5 | Junctions_alignment | *junc_align* | aligns the unmapped reads with the possible junction |
| 6 | Spanning reads | *span_reads* | extends the aligned reads to span exons thereby building transcripts |
| 7 | Report | *report* | reports the aligned reads and the potent junctions |
