# Supplementary figures and images for: PVT: An Efficient Computational Procedure to Speed up Next-generation Sequence Analysis
Source: BMC Bioinformatics. 2014 Jun 4;15:167. doi: 10.1186/1471-2105-15-167 (PMC4063226; doi:10.1186/1471-2105-15-167)

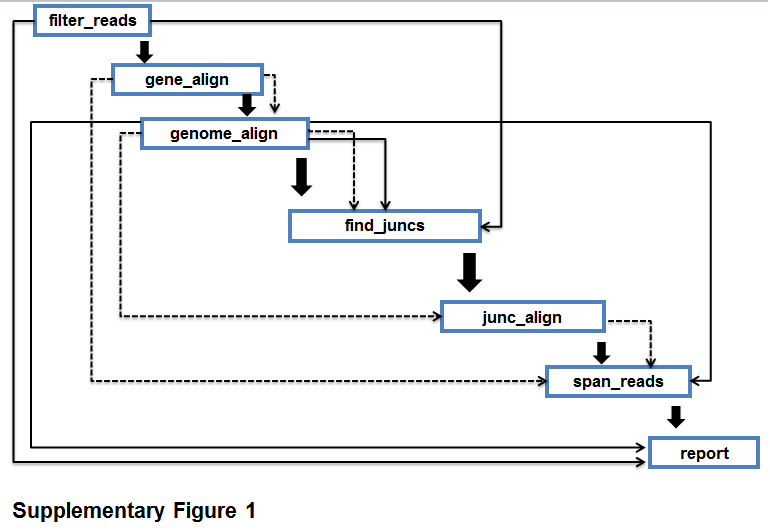

Supplement: Additional file 4: Figure S1 — TopHat pipeline and its order of execution both for single end and paired end reads ( ⇢ indicates unmapped outputs, → indicates mapped outputs). [file 1471-2105-15-167-S4.tiff]

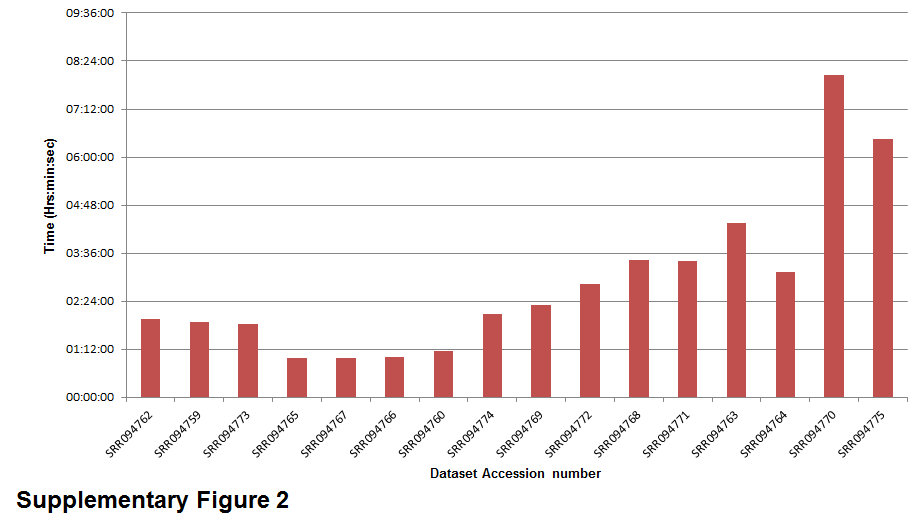

Supplement: Additional file 5: Figure S2 — TopHat Execution time corresponding to the entire single end read dataset (SRX026839 and SRX026838). [file 1471-2105-15-167-S5.tiff]

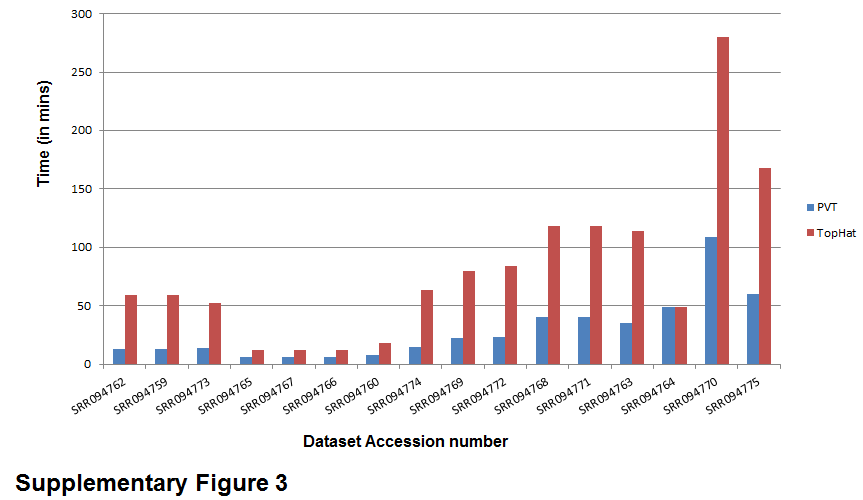

Supplement: Additional file 6: Figure S3 — Improvement of PVT over TopHat for the entire single-end read dataset in the find_juncs step. [file 1471-2105-15-167-S6.tiff]

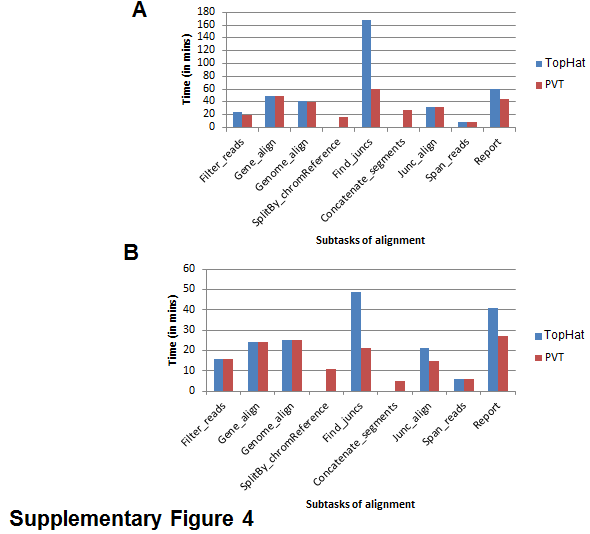

Supplement: Additional file 7: Figure S4 — Time comparison between TopHat and PVT for all the sub-steps for (A) SRR094770 (B) SRR094775. [file 1471-2105-15-167-S7.tiff]

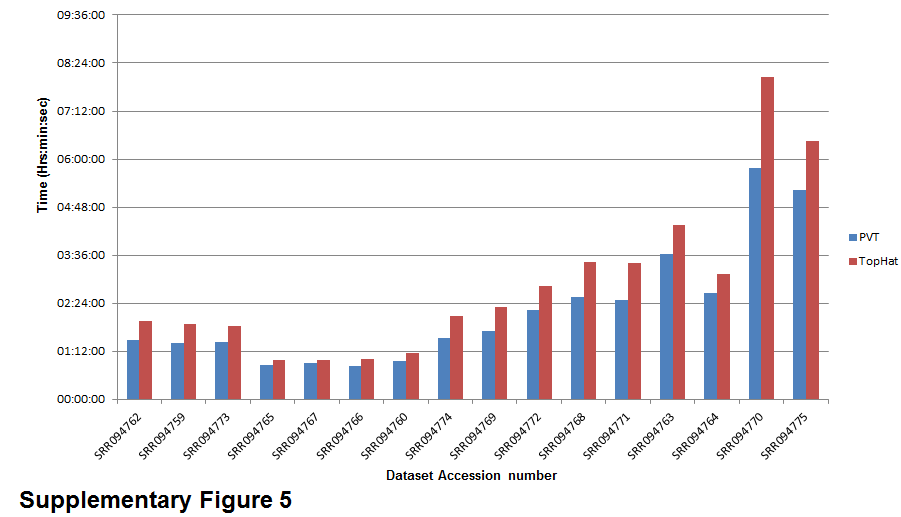

Supplement: Additional file 8: Figure S5 — Bar Graph representing the comparison of PVT execution time with that of TopHat for the entire single end read dataset. [file 1471-2105-15-167-S8.tiff]

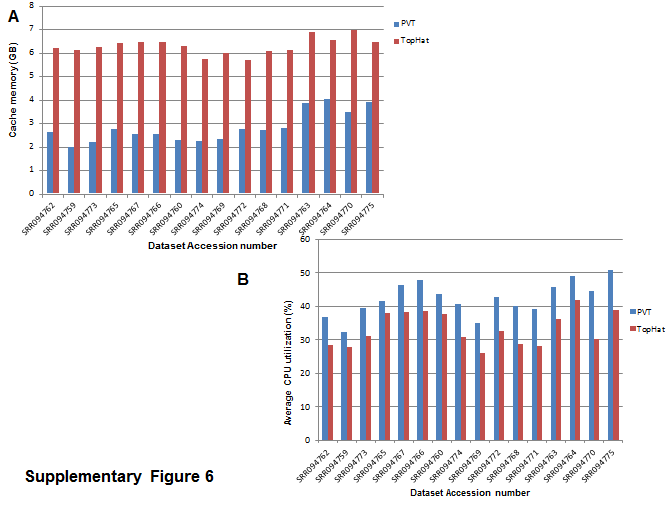

Supplement: Additional file 9: Figure S6 — Comparison of (A) Cache memory (in GB) utilization (B) average CPU utilization (in %) for the entire single end read dataset. [file 1471-2105-15-167-S9.tiff]

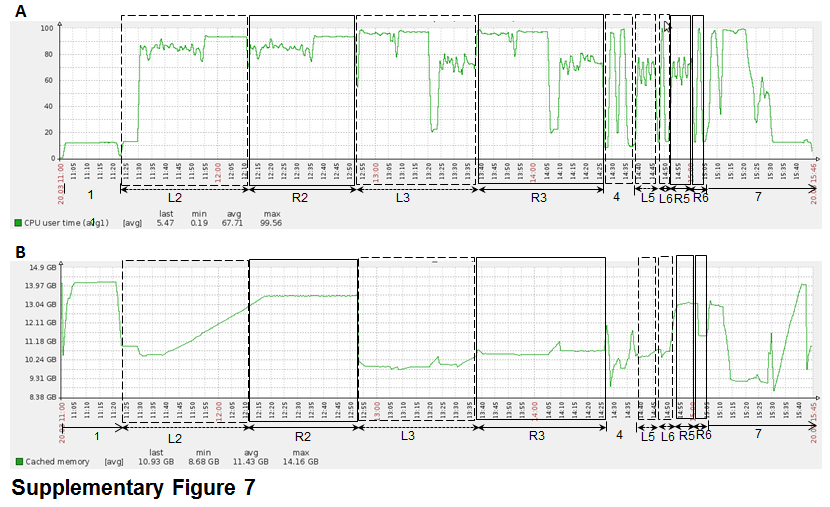

Supplement: Additional file 10: Figure S7 — Time(x-axis) vrs (A) CPU utilized (B) cache memory utilized in a standalone system with 16 GB of onboard RAM and 8 cores (3.3 GHz) CPU during the run for paired end read (SRR1027730) using TopHat. Abbreviations indicated in bold below arrow denotes the different steps of execution: L- left kept reads, R- right kept reads, numbers- denotes the step number 1: filter_reads. 2: gene_align. 3: genome_align. 4: find_juncs. 5: junc_align. 6: span_reads. 7: report. [file 1471-2105-15-167-S10.tiff]

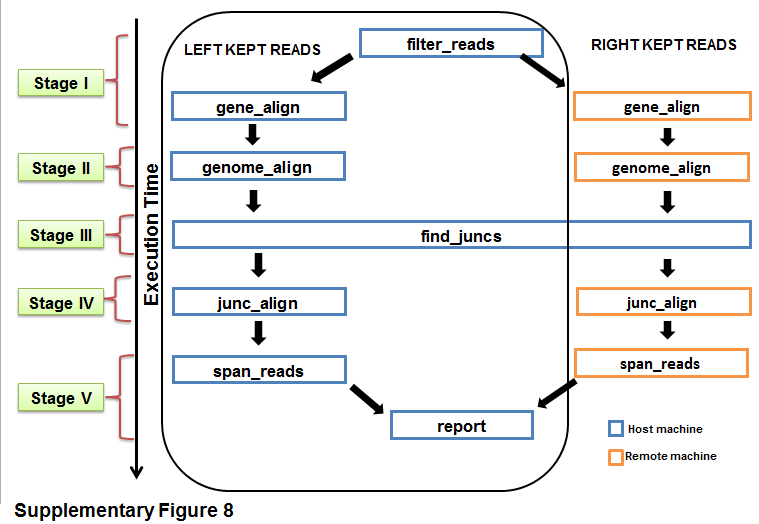

Supplement: Additional file 11: Figure S8 — PVT pipeline showing the order of execution and different stages for single end and paired end reads. The step(s) comprising each stage is based on balanced length of pipeline stage. Single end read analysis pipeline is presented within black bordered box. [file 1471-2105-15-167-S11.tiff]

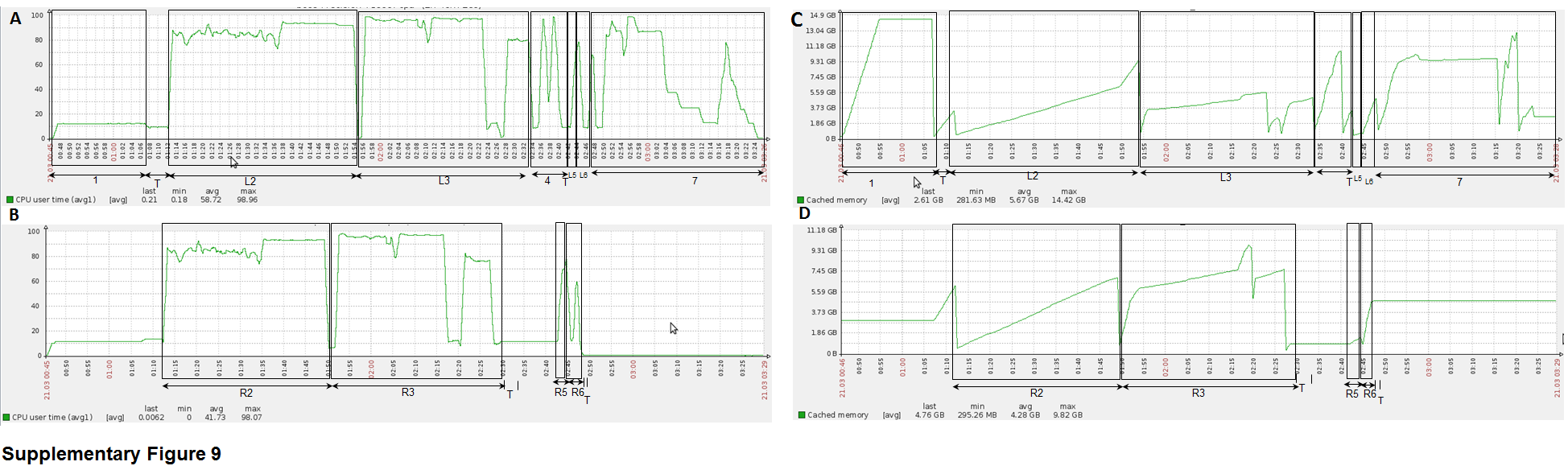

Supplement: Additional file 12: Figure S9 — Time (x-axis) vs (A) CPU utilized in the host machine (B) CPU utilized in the remote machine (C) cache memory utilized in the host machine and (D) cache memory utilized in the remote machine during the run for paired end reads (SRR1027730) using PVT. Abbreviations indicated in bold below arrow denotes the different steps of execution: L- left kept reads, R- right kept reads, numbers- denotes the step number 1: filter_reads. 2: gene_align. 3: genome_align. 4: find_juncs. 5: junc_align. 6: span_reads. 7: report. [file 1471-2105-15-167-S12.tiff]
