# Supplementary material for: PVT: An Efficient Computational Procedure to Speed up Next-generation Sequence Analysis
Source: BMC Bioinformatics. 2014 Jun 4;15:167. doi: 10.1186/1471-2105-15-167 (PMC4063226; doi:10.1186/1471-2105-15-167)
Supplement: Additional file 13: Table S4 — Spliced alignment steps corresponding to each pipeline stage. [file 1471-2105-15-167-S13.doc]

**Supplementary Table 4:**

| **Stages** | **Steps** |
| --- | --- |
| Stage I | *filter_reads* |
|  | *gene_align* |
| Stage II | *genome_align* |
| Stage III | *find_juncs* |
| Stage IV | *junc_align* |
| Stage V | *span_reads* |
|  | *Report* |
