# Supplementary material for: PVT: An Efficient Computational Procedure to Speed up Next-generation Sequence Analysis
Source: BMC Bioinformatics. 2014 Jun 4;15:167. doi: 10.1186/1471-2105-15-167 (PMC4063226; doi:10.1186/1471-2105-15-167)
Supplement: Additional file 14: Table S5 — Pseudo-code of PVT for single end read analysis. [file 1471-2105-15-167-S14.doc]

**Supplementary Table 5:**

| **PVT pipeline for single end read** |
| --- |
| PVT pipeline  {  filtered_reads = filter_reads(input_fastq) ;  clear_memory();  gene_mapped_reads, gene_unmapped_reads =  gene_align(filtered_reads);  clear_memory();  genome_mapped_reads, genome_unmapped_reads =  genome_align(gene_unmapped_reads) ;  clear_memory();  split_alignments[i] = splitBy_chromReference(genome_mapped_reads);  clear_memory();  foreach(i){  segment_juncs_seq[i] = find_juncs(genome_unmapped_reads,  split_alignments[i] ) &;  segment_seq = concatenate_segments (segment_juncs_seq[i] );    }  clear_memory();  segment_juncs = junc_align (segment_seq, genome_unmapped_reads);  clear_memory();  spliced_reads = span_reads (segment_juncs, genome_mapped_reads);  clear_memory();  accepted_alignments = report(filtered_reads , spliced_reads,  genome_mapped_reads );  } |
