# Supplementary material for: PVT: An Efficient Computational Procedure to Speed up Next-generation Sequence Analysis
Source: BMC Bioinformatics. 2014 Jun 4;15:167. doi: 10.1186/1471-2105-15-167 (PMC4063226; doi:10.1186/1471-2105-15-167)
Supplement: Additional file 15: Table S6 — Pseudo-code of PVT for paired end read analysis. [file 1471-2105-15-167-S15.doc]

**Supplementary Table 6:**

| **PVT pipeline for paired end read** |
| --- |
| PVT pipeline  {  left_filtered_reads, right_filtered_reads = **filter_reads**(input_fastq) ;  clear_memory();  check_smaller_size(left_filtered_reads, right_filtered_reads);  //assuming right_filtered_reads is smaller  transfer_to_remote(right_filtered_reads);  left_gene_mapped_reads, left_gene_unmapped_reads  =**gene_align**(left_filtered_reads);  right_gene_mapped_reads, right_gene_unmapped_reads  =**gene_align**(right_filtered_reads);  clear_memory();  left_genome_mapped_reads, left_genome_unmapped_reads =  **genome_align**(left_gene_unmapped_reads) ;  right_genome_mapped_reads, right_genome_unmapped_reads =  **genome_align**(right_gene_unmapped_reads) ;  clear_memory();  transfer_from_remote(right_genome_mapped_reads,right_genome_unmapped_reads);  segment_juncs_seq =  **find_juncs**(left_genome_unmapped_reads,right_genome_unmapped_reads);  transfer_to_remote(right_genome_unmapped_reads);  left_segment_juncs =  **junc_align**(segment_juncs_seq,left_genome_unmapped_reads);  right_segment_juncs =  **junc_align**(segment_juncs_seq,right_genome_unmapped_reads);  clear_memory();  left_spliced_reads =  **span_reads** (left_segment_juncs,left_genome_mapped_reads);  right_spliced_reads =  **span_reads** (right_segment_juncs,right_genome_mapped_reads);  clear_memory();  transfer_from_remote(right_spliced_reads)  accepted_alignments = **report**(left_filtered_reads , right_filtered_reads, left_spliced_reads, right_spliced_reads, left_genome_mapped_reads, right_genome_mapped_reads );  }  spliced_reads = span_reads (segment_juncs, genome_mapped_reads);  clear_memory();  accepted_alignments = report(filtered_reads , spliced_reads,  genome_mapped_reads );  } |
